# Impact of Weekly Community-Based Dance Training Over 8 Months on Depression and Blood Oxygen Level–Dependent Signals in the Subcallosal Cingulate Gyrus for People With Parkinson Disease: Observational Study

**DOI:** 10.2196/44426

**Published:** 2024-12-13

**Authors:** Karolina A Bearss, Rebecca E Barnstaple, Rachel J Bar, Joseph F X DeSouza

**Affiliations:** 1 Department of Psychology, Algoma University, Brampton, ON, Canada; 2 Dance Studies, York University, Toronto, ON, Canada; 3 Department of Theatre, University of Guelph, Guelph, ON, Canada; 4 Department of Creative Arts, Health and Wellness, University of Guelph, Guelph, ON, Canada; 5 Canada’s National Ballet School, Toronto, ON, Canada; 6 Connected Minds: Neural and Machine Systems for a Healthy, Just Society, York University, Toronto, ON, Canada; 7 Department of Psychology, Centre for Vision Research, Toronto, ON, Canada; 8 Graduate Program in Interdisciplinary Studies, York University, Toronto, ON, Canada; 9 Vision: Science to Applications, York University, Toronto, ON, Canada

**Keywords:** depression, cingulate, GDS, Geriatric Depression Scale, neuroimaging, dancing, Parkinson disease, neurodegenerative, MRI, imaging, neurology, magnetic resonance imaging

## Abstract

**Background:**

Dance has emerged as a complementary treatment that may promote adaptive neural plasticity while improving symptoms of Parkinson disease (PD), such as balance, gait, posture, and walking. Understanding brain changes that arise from participation in dance interventions is important as these neural plastic changes play an important role in protecting and healing the brain. Although dance has been shown to improve PD motor and nonmotor symptoms, the neural mechanisms underlying these changes, specifically depression and mood, remain elusive. Further, many side effects of PD drug treatments can be exacerbated or even induced by dopaminergic drugs, particularly depression and anxiety, making these nonmotor symptoms more noticeable throughout the progression of the disease.

**Objective:**

In this study, we focused on the impact of dance interventions on PD nonmotor symptoms by conducting an 8-month observational study, tracking the relationship between depression scores and functional neuroimaging measures for people with PD.

**Methods:**

A total of 34 dancers—23 (68%) people with PD and 11 (32%) healthy controls—completed the Geriatric Depression Scale (GDS) before and after attending weekly community-based dance classes, referred to as Dance for PD classes. Specifically, we examined changes within the functional magnetic resonance imaging signal from the subcallosal cingulate gyrus (SCG), an important node within the depression network and a controversial target for deep brain stimulation in the treatment of major depressive disorder.

**Results:**

Depression scores on the GDS decreased in each preintervention to postintervention comparison (all *P*<.025). In addition, GDS scores also improved over the 8-month dance period (all *P*<.01). Blood oxygen level–dependent signals from frontal cortex brain region implicated for emotional regulation within the SCG decreased at each testing time point (all *P*<.05). Also, a significant decrease in depression scores (GDS) was correlated with reduced blood oxygen level–dependent signals from the SCG (*P*=.02).

**Conclusions:**

This study contributes to an improved understanding of the neural mechanisms that are involved in depression, as well as the beneficial contribution that longitudinal dance interventions have in reducing nonmotor symptoms associated with PD, particularly in depression symptoms.

## Introduction

Depression affects 280 million people globally and is considered a prodromal feature for increasingly prevalent neurodegenerative conditions, including Parkinson disease (PD). More than 10 million people live with PD, the fastest growing neurological disorder worldwide [1]. Most commonly reported PD symptoms pertain to motor and nonmotor symptoms (NMSs), such as anxiety, depression, fatigue, apathy, cognitive disturbances, and dementia [2-4], significantly impacting health and quality of life (QoL). Anxiety disorders, which frequently present prior to or in conjunction with major depression [5], have been shown to be indicative of prodromal stages of PD [6] and are also associated with disease progression [7].

Despite the high incidence and impact of depression on QoL, the most prevalent treatments for PD—dopamine replacement therapy and deep brain stimulation (DBS)—target motor symptoms. Traditional pharmacological interventions for PD such as levodopa and related medications can also be accompanied by unpleasant side effects, with efficacy subjected to decay over time. Furthermore, surgical intervention with DBS is only available to suitable candidates, is not always successful, and may exacerbate some symptoms [8].

Over the past 2 decades, a growing body of research has indicated that there are extensive benefits for people with PD associated with engaging in dance, a low-cost and readily available activity with historic and cultural precedents in many cultures and communities. Dance-based programs specifically designed for this population have been shown to reduce motor impairments through improving balance and decreasing motor symptom severity, while also being linked with positive changes in NMSs such as mood [9-13] and improvements in QoL; these findings were demonstrated using the 16-item QoL Scale from the Oregon Health and Sciences University, a validated scale for assessments in persons with chronic diseases, which estimates overall QoL beyond issues only related to health. A post–dance class questionnaire developed by Westheimer [14] found well-being to be associated with dance for people with PD [15], and research from Kalyani et al [16] saw improvements in QoL on the Parkinson’s Disease Questionnaire.

Dance for PD (DfPD), a dance program designed for people with PD and used internationally, is open to participants of all ages and stages of disease. In the DfPD model, dance instructors are trained to ensure classes are appropriate and beneficial for people with PD. Participants are invited to explore movements based in ballet and contemporary dance, which are adapted to a range of motor abilities. While emphasizing artistry, creativity, and beauty, DfPD maintains the central importance of dance by treating participants not as patients but as dancers; DfPD also provides a respite from PD diagnosis and an alternate means of experiencing the body [12,14,17-19].

To date, there has only been 1 functional magnetic resonance imaging (fMRI) case study with a single participant, in which motor improvements and neural changes were explored after a 7-week dance intervention among 7 people with PD. The authors found significant improvements on the Fullerton Advanced Balance scale and putative brain network connectivity changes between the basal ganglia and premotor cortices in this 1 participant [20]. A 2021 behavioral study on dance and emotional arousal in people with PD found a significant correlation between the levels of General Positive Affect and average Skin Conductance Levels, with significantly higher Skin Conductance Level rates during DfPD classes compared to a matched-intensity exercise; this suggests specific and additional emotional benefits for dance [12]. Dance, as opposed to exercise, involves relational movements such as moving toward and away from other dancers or responding to their actions, which may elicit emotional states and memories. These aspects of social interaction are inherent to dance, and their potential influence on neural circuitry associated with mood and memory has never been examined. While many studies of dance for people with PD have shown promising behavioral results for depression or emotional changes, they are often limited by small sample sizes and a brief intervention period (weeks to a few months). Additionally, the time course and duration of these effects are unknown, particularly for NMSs such as depression. Most importantly, no study has examined emotional brain activation for a community-based dance class in people with PD longitudinally.

This observational study examined the brain-behavior interactions of emotional processing associated with learning and performing a specific dance (choreography) while attending weekly DfPD classes, as well as NMSs associated with depression, over 8 months. We hypothesized that multisensory learning associated with engaging in dance may act as a buffer against neurodegenerative disease [13], and that this would be visible through modulations in brain signals over time within the emotional circuit used for treatment-resistant depression [21,22]. Specifically, we looked at changes only within the fMRI signals from the subcallosal cingulate gyrus (SCG), an important, and still controversial, node within the depression network [21,22]. We assessed changes in the SCG using noninvasive neuroimaging for at least 2 time points over 8 months for each participant following a DfPD class, and we measured mood and depression before and after the dance class in the studio. Our findings suggest that participation in a nonmedical intervention (dance) may have the potential to modify emotional brain circuitry over time.

## Methods

### Overview

Participants that were involved in this study were recruited from weekly, 75-minute, community-based, and structured DfPD classes that were offered in Toronto at Canada’s National Ballet School. Recruitment consisted of verbal communication and explanation of the study’s interests, procedures, and other details at the start of a dance class. Participation in the study was completely voluntary and solely based off of personal interest of the participants in this research. Interested participants would then be provided with a hard copy of the study details, supplemented by verbal communication with the researchers, to help answer any questions and help them further decide whether they would like to participate. These classes begin with a seated component, followed by mirroring and paired exercises, and end with standing and executing choreographed sequences and locomoting through space (see Table 1 from Bearss et al [15]). Notably, the specific choreography involved in this study included sequences in which actions were directed toward another participant and dancers often moved in relation to a partner or the group. Participants learned and practiced the sequences at the end of each weekly class from September to June and publicly performed the choreography on 3 separate occasions within the year of fMRI: Sharing Dance Day, performances at Toronto City Hall, and a Parkinson’s Central annual meeting.

### Ethical Consideration

Written informed consent was obtained at each data collection time point throughout the entire 8-month data collection period, using an approved protocol from York University’s Ethics Board (2013‐211 & 2017‐296). All the procedures followed were in accordance with the ethical standards of the responsible committee on human experimentation (institutional and national) and with the Helsinki Declaration of 1975, as revised in 2000. Privacy and confidentiality of the participant data were protected by deidentification, and all data were stored on password-protected external hard drives at the laboratory at York University. There are no identifying data in the figures of this study. Compensation for each participant involved CAD $25 per imaging session per participant and CAD $50 reimbursement for travel to York University for imaging (CAD $1=~US $0.71).

### Behavioral Testing

A total of 34 participants volunteered from the DfPD classes at Canada’s National Ballet School: 23 (68%) people with PD and 11 (32%) healthy controls. People with PD were between the ages of 52‐76 (mean 67.78, SD 6.14) years (male participants: n=9; mean diagnosis duration: 5.56, range 0‐17 y). Healthy controls were between the ages of 61‐83 (mean 70.11, SD 7.4) years (male participants: n=6). Participants completed the Geriatric Depression Scale (GDS) before and after a single dance class (1.25 h) at 3 separate time points: March, April, and June.

### Behavioral Analysis

Data from the GDS were analyzed in SPSS statistical software (version 24.0; IBM Corp 2016), using a linear mixed effects model analysis to account for individual participant data variability and to allow for the inclusion of data from participants missing 1 or more data collection point across time (March, April, and June).

### Functional Imaging Phase

**Figure 1.**
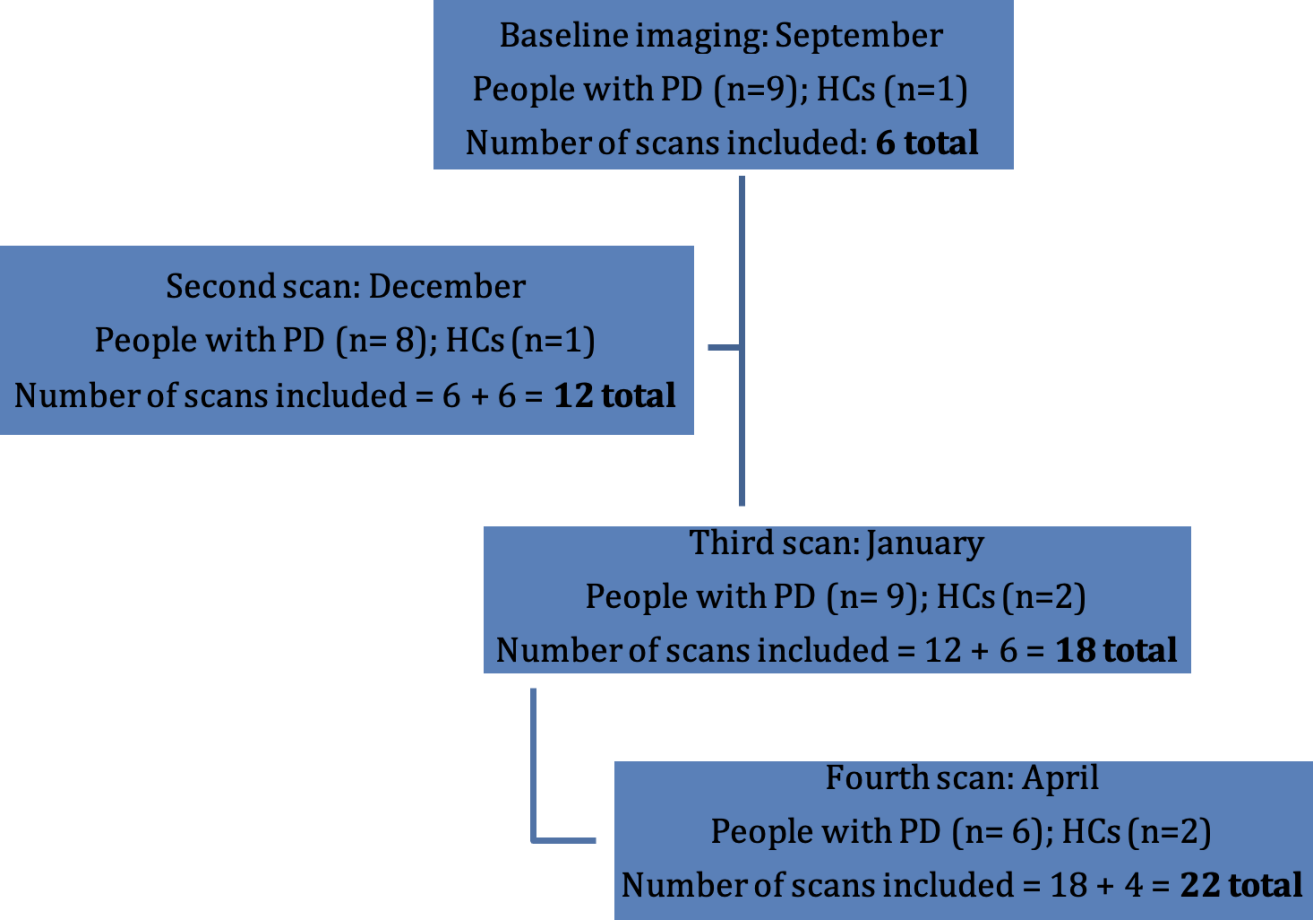
Flowchart of participant enrollment into our study over 8 months (September, December, January, and April). HC: health control; PD: Parkinson disease.

A total of 15 participants volunteered and were scanned at 1 of the 4 time points in our study (Figure 1). Unfortunately, in our longitudinal, observational, community-based dance study, 5 people with PD and 3 healthy controls did not complete the required 2 fMRI sessions or 2 sessions of the GDS. Thus, we had 7 people with PD (mean age 71.33, SD 5.51 y; male participants: n=4; mean diagnosis duration: 8.75, SD 5.75 y) complete 22 functional scans in September, December, January, and April at York University’s Sherman Health Sciences Centre (see Figure 1). Disease progression ranged from asymptomatic to severe, as measured by the Hoehn and Yahr Scale (range 0‐4; mean 0.8).

### Procedures for Functional Imaging

A 3T Siemens Tim Trio magnetic resonance (MR) scanner was used to acquire functional and anatomical images using a 32-channel head coil. T2*-weighted echo planar imaging was conducted using parallel imaging (generalized autocalibrating partially parallel acquisitions), with an acceleration factor of 2× and the following parameters: 32 slices, 56×70 matrix, 210×168 mm field of view, 3×3×4 mm voxels, echo time=30 ms, flip angle=90°, and volume acquisition time=2.0 s, for a total of 240 volumes per scan. Echo planar images were coregistered with the high-resolution (1 mm^3^) anatomical scan of the participant’s brain taken at the end of each session (spin echo, echo time=2.52 ms, flip angle=9°, and 256×256 matrix). Each participant’s head was restrained with padded cushions to reduce head movement artifacts.

While in the MR scanner, participants were instructed to visualize themselves dancing from an internal first-person perspective while listening to music associated with the choreography learned in class (for more details on this protocol, see Bar and DeSouza [23]). This learning paradigm had been developed and used as a means of probing activity within the auditory and supplementary cortex of expert professional ballerinas during the learning of dance motor sequences [23]. The brief (8-min) scanning protocol is conducive to collecting longitudinal samples and was shown to activate music and imagery networks in our previous study [23]. In this study, we applied this same protocol to probe for changes in a mood network.

The learned choreography was a total of 3 minutes in duration; however, only the first minute of music (Aaron Copeland’s “Hoe-Down”) was used during the fMRI, as this was the material learned at the first (September) scan. The dance visualization task used a blocked design of 30 seconds *off* and 60 seconds *on*; *on* states were alternated 5 times with 30-second periods of rest for a total scan time of 8 minutes. Our group developed this paradigm in 2011 as a means to probe changes associated with learning choreography in the dance studio, which can then be imagined and rehearsed in the MR scanner while being scanned [23]. Our seed region was set using anatomical landmarks from DBS studies used to alleviate depression in major depressive disorder by Mayberg et al [22,24], with a surrounding 5-cm radius in BrainVoyager 22.0 (v22.0.3.4578; Brain Innovation) to sample our participants’ blood oxygen level–dependent (BOLD) signals during 4 periods of learning dance choreography in the studio [25].

### Preprocessing

Functional scans were superimposed on anatomical brain images, aligned on the anterior commissure–posterior commissure line, and transformed into Talairach space in BrainVoyager 22.0. Functional data from each scan were screened for motion and/or magnet artifacts to detect abrupt movements of the head. In addition, we ensured that no obvious motion artifacts were present in the activation maps from individuals with PD. Participants were video recorded while in the MR scanner to monitor for possible physical movements during scanning. None of the 22 functional scans from the 7 people with PD were removed due to motion artifacts, with none of the scans exceeding 2-mm in-plane during the motion correction algorithms.

### Statistical Analysis

Following statistical analysis of the BOLD signals, data analysis was conducted in MATLAB (version 9.8.0.1417392, R2020a; The MathWorks Inc.) and RStudio (version 1.2.5033; Posit). We used an anatomically defined seed region (radius of 5 mm) corresponding with the coordinates of the SCG, as modulation of activity in this area has been associated with changes in depression and mood [22,24]. Behavioral and functional data were analyzed using a Pearson correlation analysis of the change in GDS data from before to after fMRI and the decrease in BOLD signals over the 2 time points collected.

## Results

### Behavioral Phase in the Community Setting

The linear mixed effects model analysis showed improvements in depression and mood scores when examining dance participation over time. There was a significant main effect for time *(F*_4,135.31_=3.677, *P*<.01) and the measured effect of the dance class on the GDS score (before vs after; *F*_1,125.97_=5.266, *P*<.025); no significant interaction was found between experience (time in months dancing) and the impact on the GDS score following a 75-minute dance class *(F*_2,125.85_=0.166, *P*=.85).

**Figure 2.**
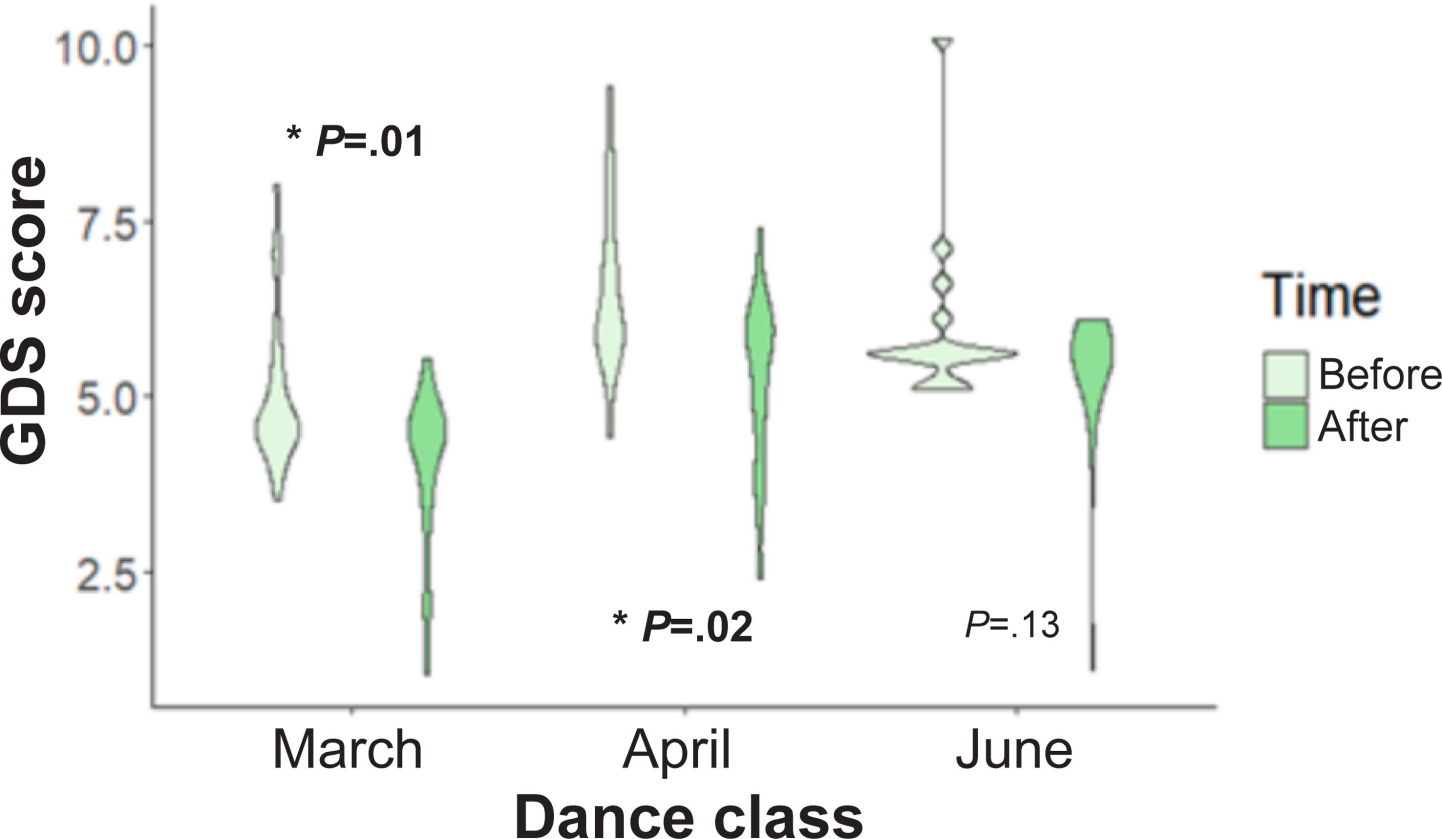
Mean Geriatric Depression Scores (GDS) scores across all dance participants (23 people with PD and 11 healthy controls) before and after a dance class conducted in the dance studio in March, April, and June. *P<.05. PD: Parkinson disease.

Figure 2 shows a significant reduction of GDS scores across time before versus after the dance class for March (*P*=.007) and April (*P*=.02), as well as across all months of data collection. Further examination of the before versus after class comparisons used paired 1-tailed *t* tests to examine the significance of the conditions (before vs after) across the differing time points and found a significant reduction in reported GDS symptoms. Significant differences in GDS scores were observed among people with PD when examining GDS scores for before versus after class at each of the time points, as well as looking at the condition between specific time points.

### Functional Neuroimaging Phase

**Figure 3.**
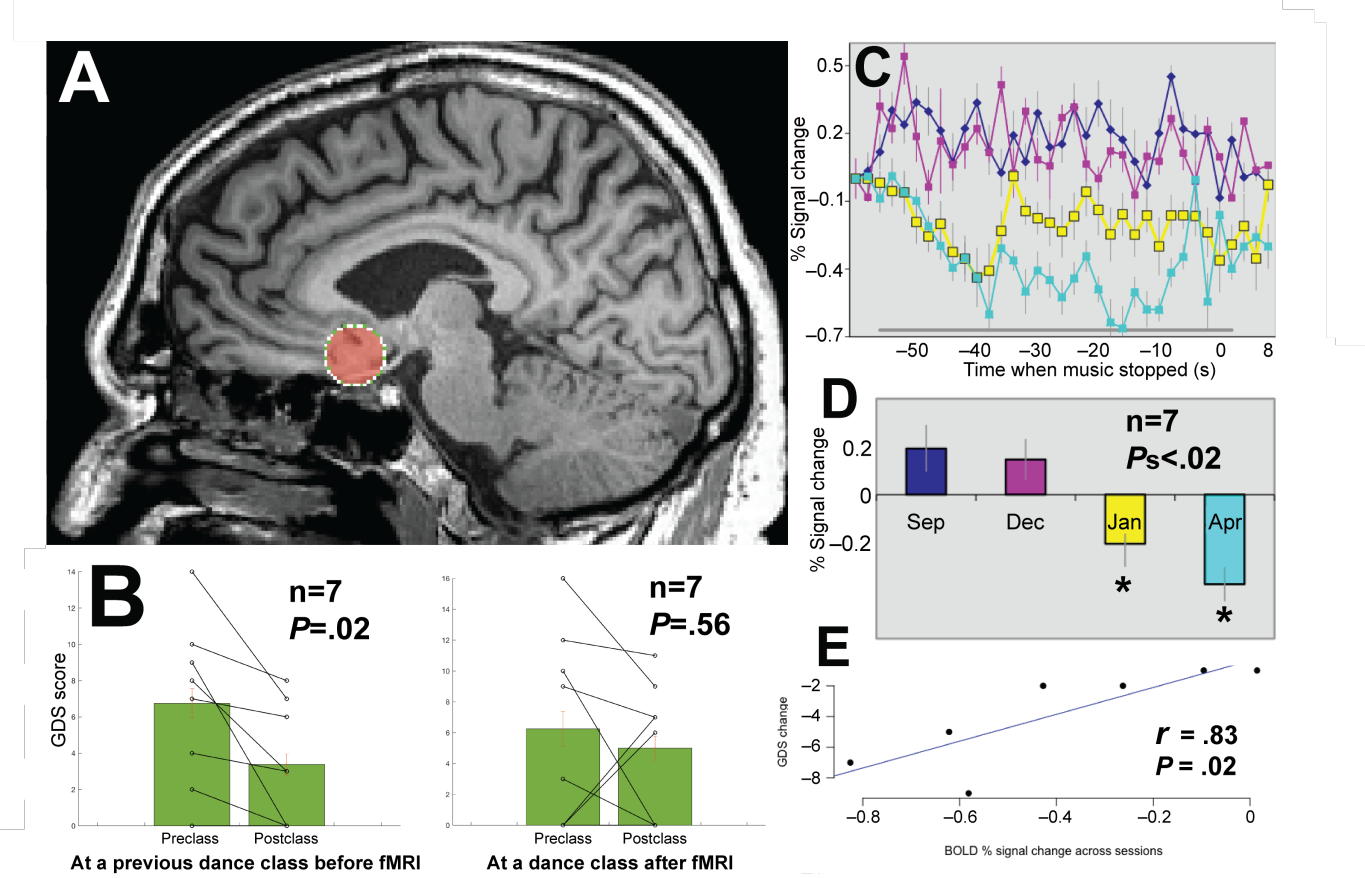
(A) Anatomical location of the seed in the frontal cortex node of the subcallosal cingulate gyrus (SCG) to probe emotional BOLD signals while people with PD imagined or visualized learned choreography elicited by the music used in the DfPD class. (B) Geriatric Depression Scale (GDS) scores for this subset of people with PD (*P*<.05; paired 1-tailed *t* test; n=7). (C) BOLD signals averaged across 7 people with PD for the 1 minute of music and the imagery or visualization training task during September, December, January, and April (22 scans). Data are aligned to the end of the 1 minute of music. (D) Bar graphs showing averaged BOLD signals from the SCG with SE of the mean; asterisks (*) signify a significant difference from the September scan. (E) A significant correlation of GDS scores and BOLD signals for the 7 people PD who both underwent the fMRI and completed the GDS questionnaires at the dance studio. BOLD: blood oxygen level–dependent; DfPD: Dance for Parkinson Disease; fMRI: functional magnetic resonance imaging; PD: Parkinson disease.

Based on literature showing the SCG to be a crucial node in affective emotional networks associated with major depression disorder, an anatomical seed localizer for this brain region (Figure 3A) was used in extracting BOLD signals from 7 dancers with PD who had overlapping GDS scores collected during the sampling periods. Figure 3B shows the GDS scores for a subset of 7 people with PD who were scanned in the MR scanner at appropriate times and filled in questionnaires at 2 points in the community dance class. Figure 3C shows a decrease in the averaged signal across the 1 minute of music where the people with PD visualized or imagined their learned dance at 4 different moments over the learning process (September, December, January, and April). Figure 3D shows the BOLD signal pattern decrease from September to sessions in January and April (*P*=.02 and *P*=.005, respectively). A Pearson correlation analysis of the change in GDS score and the change in BOLD signal showed a significant correlation (*r*=.83; *t*_5_=−3.31, *P*=.02; Figure 3E).

## Discussion

### Overview

Depression, in its many potential manifestations, can be a serious and debilitating disorder, impacting every aspect of life and decreasing QoL for people with PD. It is also incredibly common; in 2020, an estimated 6% of all US adults had at least 1 major depressive episode [26]. Despite the prevalence of depression, the neural mechanisms underlying its development, expression, and treatment remain poorly understood. As an example of the complexity of this subject, the commonly held hypothesis that low serotonin levels are linked with depression is now under debate, emphasizing the need to develop and further investigate novel treatments [27]. Our research contributes to initial evidence that participating in a social activity such as dance can alter activity in a brain region associated with depression and mood.

### Principal Findings

Longitudinal reductions in BOLD brain signals from the SCG, an area believed to be a critical node in a corticolimbic network associated with depression [24], correlated with decreases in GDS depression scores during our dance program in people with PD. This observational study combines local data collected at the intervention site (dance studio) with functional neuroimaging. In the MR scanner, participants mentally rehearsed learned movement patterns associated with their in-studio choreographed dance sequences while listening to the music that accompanies the dance. The reduction in BOLD activation observed in a region involved in depression provides preliminary evidence that participation in dance classes involving learned choreography can modulate neural activity in a way that is adaptive.

### Comparison to Prior Work

Many imaging studies suggest increased activity in the SCG region for patients with depression [22,24] and reductions in activity following treatment with antidepressants, placebos, repetitive transcranial magnetic stimulation, electroconvulsive therapy, DBS, and cingulotomy [21,24]. However, none of these studies were conducted with people with PD or used dance as the intervention. Our investigation provides initial evidence that brain signals from the SCG decrease in people with PD who participate in noninvasive, weekly, community-based dance classes over a period of 8 months while learning specific movements of their bodies in a community space, moving through space in synchrony with others in the studio.

The SCG plays an important role in regulating emotion. Located in the subgenual anterior cingulate cortex, degeneration in this area correlates with depressed mood and anhedonia [28]. Specifically, the SCG is thought to be a critical node in regulating a corticolimbic network, with increased activity in this area associated with depression [22]. Also, the SCG is a primary target in DBS for treatment-resistant depression [29]. It is hypothesized that DBS can modulate the depression network through a reduction of SCG activity [22,24], leading to antidepressant effects.

### Strengths and Limitations

In the wake of COVID-19, there has been a global increase in mental health concerns closely associated with reduced access to social activities and emotional support. Group movement experiences associated with health, art, and ritual have played a role in human culture for millennia. Beyond their aesthetic value, we showed [12] that these can play an important role in regulating mood and adaptive behaviors. Our participants showed a reduction of BOLD signal activity in the SCG over the 8-month period, during which they participated in weekly group dance sessions that involved learning a new motor sequence set to music, an experience that was re-elicited in the MR scanner.

Depression is one of the most prevalent and debilitating NMSs faced by people living with PD and may even precede motor disturbances. It is also one of the most challenging to identify and treat because psychomotor slowing and blunted affect (masking) can contribute to missed diagnosis of depression. Importantly, interactions between some antidepressants (selective serotonin reuptake inhibitors) and PD medications, in some cases, may potentially exacerbate motor symptoms. With increased prevalence of depression across a wide variety of populations, including people with PD, new treatments and models are sorely needed, and dance [30] is emerging as an enjoyable and potentially effective option for this population.

Our study asked volunteers to participate in a new dance class associated with this research held at Canada’s National Ballet School in Toronto, an ecologically valid setting as these are the spaces in which community dance experiences occur. However, this setting also raises limitations in the application of our results, which must be interpreted with caution, as this observational study did not include a comparable control group. Future studies including people with PD who do not engage in dance will help prevent biases in the sample through a randomized controlled trial design; thus, this study must be considered as only a preliminary report of findings observed in a community setting.

### Future Directions

The match we found between biological (fMRI) and behavioral measures (GDS) provides early evidence that participation in a program such as DfPD, a noninvasive, widely available intervention throughout the world, can facilitate adaptive plastic changes in a neural network node associated with depression. This is an important finding in that it demonstrates how sociocultural art experiences may be integrated into or affect biological neural circuits. These preliminary observational results require further investigation, ideally through a randomized controlled trial to better elucidate the contribution of complex behavioral neural interactive mechanisms and reduce the potential confoundings that accompany any observational study like ours.

## Abbreviations

BOLD: blood oxygen level–dependent
DBS: deep brain stimulation
DfPD: Dance for Parkinson Disease
fMRI: functional magnetic resonance imaging
GDS: Geriatric Depression Scale
MR: magnetic resonance
NMS: nonmotor symptom
PD: Parkinson disease
QoL: quality of Life
SCG: subcallosal cingulate gyrus
